# The Complexity and Fuzziness of Conformational Chemistry: Theory and Applications

**DOI:** 10.3390/molecules31213886

**Published:** 2026-10-28

**Authors:** Pier Luigi Gentili, Vladimir N. Uversky

**Affiliations:** 1 Department of Chemistry, Biology, and Biotechnology, Università Degli Studi di Perugia, Via Elce di Sotto 8, 06123 Perugia, Italy; 2 Department of Molecular Medicine and USF Health Byrd Alzheimer’s Research Institute, Morsani College of Medicine, University of South Florida, 12901 Bruce B. Downs Blvd., Tampa, FL 33612, USA

**Keywords:** conformational chemistry, molecular complexity, complex systems, maximum entropy method, photochromism, proteins, DNA, RNA, fuzzy sets, chemical artificial intelligence

## Abstract

Molecular structure is traditionally described in terms of chemical composition, atomic connectivity, and three-dimensional geometry. However, many molecules, particularly biomacromolecules, exist not as single structures but as dynamic ensembles of interconverting conformers whose populations depend on the surrounding physicochemical context. This work proposes that such conformational ensembles can be interpreted as molecular fuzzy sets, thereby establishing a conceptual bridge between conformational chemistry, information theory, and fuzzy logic. The theoretical framework is introduced by representing molecular structures as adjacency matrices and extending this description to conformational distributions. Experimental evidence is discussed through the photochromic behavior of merocyanines, whose temperature- and environment-dependent conformational distributions encode measurable amounts of fuzzy information. Further support is provided by proteins, DNA, and RNA, where conformational heterogeneity underlies multifunctionality, gene regulation, alternative splicing, molecular recognition, and adaptive cellular responses. Within this perspective, biological function emerges from context-dependent distributions of molecular states rather than from unique static structures. Conformational ensembles are also described as thermalized quantum mixed states, suggesting new links between molecular complexity, fuzzy information processing, and quantum biological phenomena. Finally, the implications of molecular fuzziness for Chemical Artificial Intelligence are discussed, highlighting the possibility of exploiting molecular conformational dynamics as a substrate for adaptive, context-sensitive, and chemically embodied information processing.

## 1. Introduction

Life is arguably the most remarkable emergent phenomenon exhibited by certain Complex Systems. From a chemical point of view, all living beings are made of lipids, DNA, RNA, proteins, water, salts, sugars, and so on. Taken separately, these compounds can never show life. Only when all those compounds are assembled in that peculiar structural and functional spatiotemporal organization that is a cell can life emerge [1,2,3,4,5,6,7,8,9,10]. Life can be described as a sequence of temporary events that starts with birth and continues with growth, usually reproduction, aging, and, finally, death. During their life cycle, living systems exhibit adaptive and goal-directed behaviors that enable them to respond to environmental challenges and maintain functional organization. Cells can reach their fundamental goals by exploiting molecules, macromolecules, and energy-fueled physicochemical events to encode, collect, store, process, and communicate information. The basic information required for survival is stored in DNA (or RNA in RNA viruses, viroids, and virusoids), and the genome can be regarded as an information-storage and information-processing system whose activity, mediated through complex interactions with proteins, RNA molecules, and epigenetic modifications, enables adaptation and evolution [11,12].

Cells represent prototypical examples of Complex Systems [13]. Although numerous scientists have been investigating Complex Systems for many decades (one of the first seminal articles on Complexity was Weaver’s “Science and complexity” [14], written a few years after the end of the Second World War), and the label of “Complexity Science” became official in 1984 with the foundation of the American private research center “Santa Fe Institute”, a universally accepted definition of a Complex System is still lacking. The extensional definition of Complex Systems enumerates some systems that everybody would agree to include in the set of Complex Systems. If a single cell is the chemical prototype of a Complex System, then other representative examples surely are multicellular organisms, their societies, natural ecosystems, urban areas, the world economy, and the climate. The persistent difficulty in identifying a universally accepted definition of complexity suggests that complexity is not a binary attribute but rather a graded property. 

The set of Complex Systems may be interpreted as a multivariable fuzzy set that includes numerous systems, each with a different degree of membership [13]. A fuzzy set is different from a classical Boolean set because it breaks the law of excluded middle: an element may belong to a fuzzy set with a certain degree, which can be any real number included between 0 and 1 [15]. The variables that define the “Complex Systems” fuzzy set are: (1) the number of elements representing the system’s multiplicity; (2) the diversity of elements; (3) the temporal variability of the elements’ behavior; (4) the number of links connecting the elements, referred to as the system’s interconnection; (5) the diversity of links; (6) the temporal variability of links; and (7) the degree of integration among elements and links that gives rise to distinctive emergent properties at the level of the whole system, namely integration [16,17] whereby “the whole is greater than the sum of its parts”. All these variables, which characterize the system’s elements, links, and especially their integration, are influenced by the context in which the system is embedded. The degree to which a system belongs to the multivariable “Complex System” fuzzy set increases with greater multiplicity, diversity, and variability of its elements, as well as with higher interconnection, link diversity, link variability, and, most importantly, integration. Accordingly, the complexity of any system can be described as a context-dependent function of these seven variables. This definition of system complexity is dependent on the chosen level of description (e.g., molecular, cellular, or organismal) and encompasses both synchronic and diachronic dimensions of complexity, since it accounts for the structural organization of a system at a given moment as well as its evolution over time. 

In general, Complex Systems may be viewed as networks operating far from thermodynamic equilibrium and exhibiting emergent properties [10,16]. Surprisingly, these characteristics are also inherent to molecules when they are not at equilibrium. Consequently, molecules can be regarded as miniature instances of Complex Systems. Among molecules, those that are conformationally flexible are particularly suitable for investigating complexity and fuzziness. Rather than existing in a single well-defined structure, many molecules populate ensembles of interconverting conformations whose relative abundances depend on environmental conditions. The properties and reactivities of such molecules are often determined not by a single structure, but by the distribution of conformational states and the transitions occurring among them. This article examines conformational chemistry from the perspectives of complexity science and fuzzy logic. We argue that conformational ensembles can be interpreted as molecular fuzzy sets and that conformational dynamics represent a fundamental mechanism through which molecules process information. By integrating concepts from chemistry, systems theory, and information science, this framework provides new insights into biomolecular function and suggests novel applications in emerging fields such as Chemical Artificial Intelligence.

## 2. The Structural Complexity of Molecules

Taken alone, any molecule can be represented as a three-dimensional network in which atoms constitute the nodes, while covalent bonds serve as the primary edges. Non-covalent, through-space forces, such as electrostatic interactions, hydrogen bonds, and van der Waals interactions, constitute the secondary edges that complete the structural topography of the network. Accordingly, the structure of a molecule is completely specified when the following information is known:

1. The identity and abundance of the chemical elements composing the molecule, as defined by its molecular formula.
2. The pattern of covalent bonds connecting the atoms, as defined by its structural formula.
3. The three-dimensional arrangement of the atoms and the electrostatic interactions operating between them through space.

For a molecule containing N atoms, all this information can be encoded in a square adjacency matrix of the form [18,19]:

(1) $${\bar{A}}_{3D}=\left(\begin{array}{ccc} a_{1,1} & \cdots & a_{1,N}\\ \vdots & a_{i,j} & \vdots \\ a_{N,1} & \cdots & a_{N,N} \end{array}\right)$$

The matrix element $a_{ij}$ is equal to zero when atoms *i* and *j* are neither covalently connected nor sufficiently close in space to interact significantly through electrostatic forces. In all other cases, $a_{ij}\neq 0$. The informational content of the matrix is enhanced when the nonzero elements quantify the strength of the covalent bonds or noncovalent interactions that they represent. Molecules possessing the same molecular formula and atomic connectivity, corresponding to features (1) and (2) above, but differing in the spatial arrangement of their atoms, are termed *stereoisomers*. Although an infinite number of geometrical arrangements are mathematically possible, only a finite subset corresponds to minima on the molecular potential-energy surface. Depending on the energetic requirements for interconversion, stereoisomers can be divided into two broad classes. When interconversion requires bond breaking and reformation, typically involving energies of a few electron volts, the stereoisomers are classified as *configurational isomers*. In contrast, when interconversion occurs through rotations around single bonds or related structural rearrangement, and the associated energy barriers are comparable to or only moderately greater than the thermal energy available at room temperature (kT), the stereoisomers are referred to as *conformational isomers* or *conformers*.

Conformers continuously interconvert through thermally driven molecular motions. The rate of interconversion depends on the height of the free-energy barriers separating the conformational states: the lower the barrier, the faster the exchange. When the barriers are close to the available thermal energy, conformational transitions may occur on femtosecond to picosecond timescales. Conversely, steric constraints, intramolecular hydrogen bonding, disulfide bonds or other rigid covalent cross-linking, highly viscous environments, and low temperatures can substantially reduce conformational mobility and slow exchange dynamics. Furthermore, in aqueous media and polar solvents, conformational dynamics of molecules can be further restricted by the hydrophobic interactions that drive the compact packing of nonpolar groups away from water (i.e., promote hydrophobic collapse).

A compound existing as an ensemble of conformers can therefore be described by a set of adjacency matrices ${\left({\bar{A}}_{3D}\right)}_{k}$, each associated with a weighting coefficient $w_{k}$ that represents the relative abundance of the corresponding conformer [18]:

(2) $${\bar{\Gamma }}_{3D}=\left(w_{1}{\left({\bar{A}}_{3D}\right)}_{1},\;w_{2}{\left({\bar{A}}_{3D}\right)}_{2},\ldots ,\;w_{k}{\left({\bar{A}}_{3D}\right)}_{k},\ldots ,w_{N_{C}}{\left({\bar{A}}_{3D}\right)}_{N_{C}}\right)$$

The weighting coefficients satisfy the normalization condition:

(3) $$\sum_{k=1}^{N_{C}}w_{k}=1$$

Within this framework, the identity of a compound is more appropriately represented by its conformational ensemble under a specific physicochemical context (pcc). The physicochemical context encompasses the chemical and physical characteristics of the surrounding environment, including its qualitative and quantitative chemical composition (CC), temperature (T), pressure (P), and local electric (E) and magnetic (B) fields, together with their spatial $\left(x,y,z\right)$ and temporal $\left(t\right)$ dependence. Accordingly, the physicochemical context can be expressed as the multivariable function:

(4) pcc=f(CC,T,P,E,B,x,y,z,t)

If all variables defining the physicochemical context are spatially uniform, the environment is homogeneous and the compound is characterized by a single conformational distribution. However, when one or more variables vary across space, the environment becomes heterogeneous. Under such conditions, multiple local physicochemical contexts may coexist (${pcc}_{1}$, ${pcc}_{2},$…,), each associated with a distinct conformational distribution.

In practice, the matrices appearing in Equations (1) and (2) can be derived from experimentally determined or computationally generated molecular structures. For small molecules, atomic coordinates from X-ray crystallography, NMR spectroscopy, or quantum-chemical calculations can define both covalent connectivity and relevant through-space interactions. For larger biomolecules, structural information may be obtained from X-ray crystallography, cryo-electron microscopy, NMR spectroscopy, or molecular dynamics simulations. The weighting coefficients $w_{k}$ associated with the individual conformers can be estimated from experimentally observed populations or from statistical-mechanical models. In particular, if the conformers are in thermodynamic equilibrium, their relative abundances may be approximated by Boltzmann weights,

(5) $$w_{k}=\frac{exp\left(-G_{k}/RT\right)}{\sum_{k}exp\left(-G_{k}/RT\right)}$$

where $G_{k}\;$ is the Gibbs free energy of conformer k. Molecular dynamics simulations performed under different physicochemical conditions can likewise be used to estimate conformational populations and their dependence on temperature, solvent composition, crowding, ionic strength, or other environmental variables. Consequently, Equations (1)–(4) provide a practical framework for connecting experimentally or computationally accessible molecular structures to the broader concept of molecular complexity developed throughout this work.

Living cells are intrinsically (micro)heterogeneous systems [20]. This indicates that instead of being uniform “bags of soup” (i.e., uniform, well-mixed solutions), they are highly organized, compartmentalized biochemical units containing various microscopic sub-compartments, dense, molecularly crowded environments, and distinct local microenvironments, where voltage, ions, pH levels, and signaling molecules vary sharply across short microscopic distances, allowing localized regulation rather than global averages. As a consequence, a biomolecule may encounter multiple local physicochemical contexts during its lifetime, resulting in different conformational populations and, potentially, different functional behaviors. These contexts can be characterized by fluorescence microscopy, super-resolution methods, fluorescence-lifetime imaging microscopy (FLIM), Förster resonance energy transfer (FRET), single-particle tracking, and cryo-electron tomography.

Moreover, the weighting coefficients $w_{k}$ appearing in Equation (2) need not depend exclusively on the current physicochemical context. They may also reflect the previous states visited by the molecule and the sequence of environments through which it has evolved. In this sense, the conformational distribution observed under a given set of conditions can retain a dependence on past history. Such path dependence constitutes a form of molecular memory that may contribute to information storage and processing in biological systems [21].

## 3. Conformational Distributions as Molecular Fuzzy Sets

Any chemical compound that exists as a conformational distribution can also be described as a thermalized quantum mixed state (i.e., a specific type of quantum state that describes a physical system when it reaches thermal equilibrium with its surroundings or behaves as if it has) represented by the density operator ρ [22]:

(6) $$\rho =\sum_{k=1}^{N_{C}}w_{k}\left.|\psi_{k}\right\rangle \left\langle \psi_{k}|\right.$$

where each conformer is associated with a quantum state $\left.|\psi_{i}\right\rangle$, and the coefficient $w_{k}\;$ represents the probability of finding the molecule in that conformational state. The sum of the coefficients $w_{k}$ with $k=1,\ldots ,N_{C}$ is equal to 1 (i.e., $\sum_{k=1}^{N_{C}}w_{k}=1$). 

Any conformational distribution may be represented mathematically by a density operator describing an ensemble of conformational states. In the absence of quantum coherences, this representation reduces to a classical probability distribution over conformers. This formalism is useful because it naturally connects quantum wavefunctions, probabilistic mixtures, and fuzzy descriptions of molecular properties. Any conformational distribution can also be regarded as a *molecular fuzzy set* (i.e., a system that does not fit into rigid, binary “either/or” categories), because it partitions and grades physicochemical properties in a context-dependent manner. To establish this connection, it is useful to briefly recall some fundamental concepts of fuzzy-set theory and fuzzy logic.

**Figure 1:**
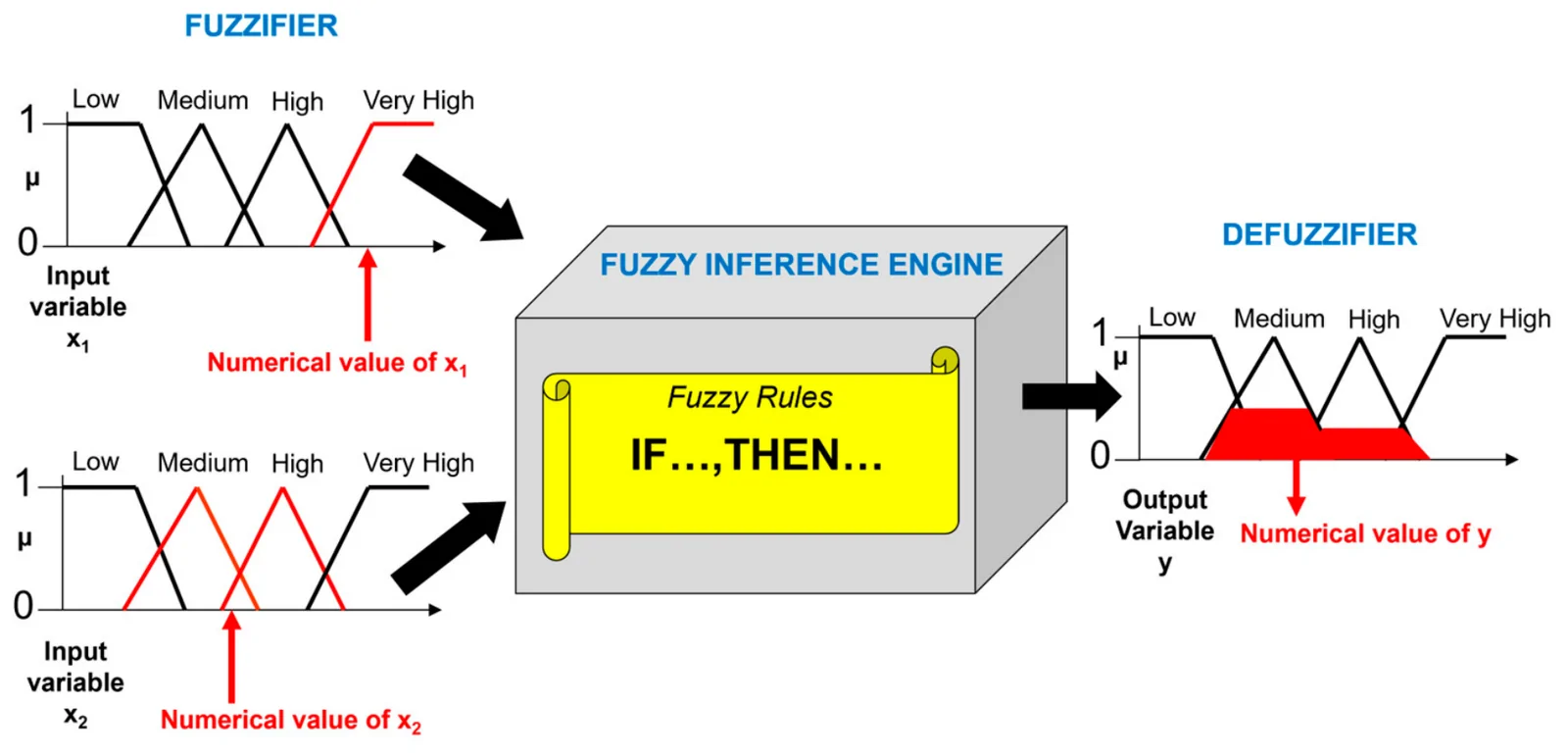
Schematic structure of a Fuzzy Logic System based on Mamdani’s method, wherein the three principal components are the Fuzzifier, the Fuzzy Inference Engine, and the Defuzzifier. The arrows indicate the information flow from the numerical values of the input variables to the numerical value of the output.

A fuzzy set has already been defined in the Introduction. In addition, it should be emphasized that fuzzy sets constitute the basic computational units of fuzzy logic. Fuzzy logic has been described as the logic of approximate or vague reasoning because it formalizes the human ability to draw conclusions from imprecise, qualitative, and linguistically expressed information [23]. Rather than operating exclusively with precise numerical values, fuzzy logic enables computation with information expressed through linguistic categories such as *low*, *medium*, *high*, or *very high*. Any complex nonlinear relationships between input and output variables can be modeled by building a Fuzzy Logic System (FLS) that relies on syllogistic statements of the type “IF…, THEN…” called fuzzy rules [24]. The fuzzy rules use adjectives for the input and output variables. Adjectives are fuzzy sets that granulate and graduate the numerical values of both the input and output variables. In this way, continuous numerical domains are transformed into overlapping qualitative categories characterized by different degrees of membership. As an example, Figure 1 reports the schematic structure of an FLS involving two input variables and one output variable. Each variable is granulated in four fuzzy sets, which are defined by the adjectives (linguistic labels) “low”, “medium”, “high”, and “very high”. In a Mamdani-type FLS [25], three fundamental components are present:

1. *Fuzzifier:* converts numerical input values into degrees of membership in the corresponding input fuzzy sets;
2. *Inference Engine*: evaluates the fuzzy rule base and determines the activation levels of the output fuzzy sets;
3. *Defuzzifier*: transforms the resulting activated output fuzzy sets (the red patches appearing in Figure 1) into a single numerical output value that can be used for prediction, classification, or process control.

The effectiveness of fuzzy logic in managing complex and unstable systems has been demonstrated by numerous engineering applications. A notable example is the stabilization of the inverted double pendulum, a highly nonlinear and dynamically unstable system that has been successfully controlled using dedicated fuzzy-logic hardware [26]. Living organisms continuously operate in environments that are no less complex, variable, and unpredictable than such engineering systems. It is therefore reasonable to hypothesize that survival and adaptation rely, at least in part, on information-processing mechanisms that are functionally analogous to fuzzy-logic operations across multiple hierarchical levels, ranging from molecules and cells to tissues, organs, and entire organisms [27]. The focus of the present work is the molecular level (for the cellular and organ levels, check references [24,28,29]). Specifically, we propose that a chemical compound behaves as a molecular fuzzy set whenever it exists as an ensemble of interconverting conformers or/and experiences distinct microenvironments characterized by peculiar physicochemical contexts. The following two subsections (Section 3.1 and Section 3.2) provide experimental evidence supporting this interpretation and illustrate how conformational distributions enable molecules to function as natural fuzzy information-processing systems.

### 3.1. Experimental Evidence Supporting the Fuzzy-Set Description of Merocyanine Conformers

**Figure 2:**
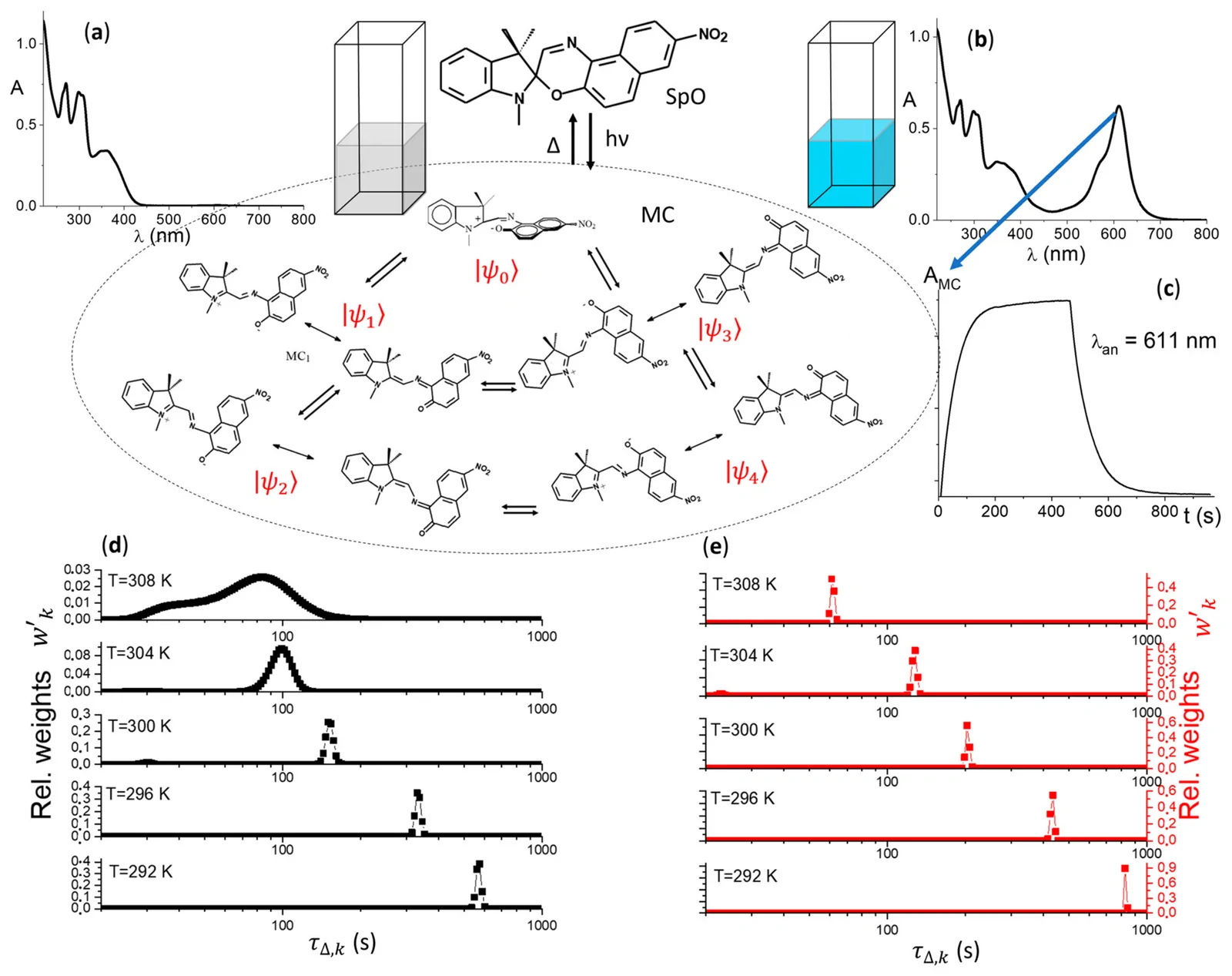
The photochromic behavior of a spiroxazine (SpO) that absorbs in the UV (see spectrum (**a**)) and gives rise to a merocyanine (MC) that also absorbs in the visible region (spectrum (**b**)) and can exist in many conformers. A few conformers are shown in the center of the figure, embedded within a dashed ellipse. Plot (**c**) shows the trend of the absorbance at 611 nm during a photochromic cycle. Plot (**d**) displays the lifetimes’ distribution for MC dissolved in pure ethanol and at different temperatures. Plot (**e**) depicts the same lifetimes’ distribution but in the presence of glycine.

Experimental evidence supporting the interpretation of conformational distributions as molecular fuzzy sets is provided by the merocyanine (MC) shown in Figure 2. MC is generated by ultraviolet irradiation of the spirooxazine (SpO) depicted at the top center of Figure 2. SpO is a direct photochromic compound [30] that is essentially colorless in solution, as indicated by the absorption spectrum shown in Figure 2a. Upon UV excitation, cleavage of the spiro C-O bond occurs, producing the corresponding merocyanine form. Owing to the extension of π-electron conjugation over the molecular skeleton, MC exhibits a pronounced absorption band in the visible region (Figure 2b) and appears blue when dissolved in ethanol. The merocyanine possesses a highly flexible molecular structure and can therefore adopt multiple conformations [31,32,33], some of which are illustrated in the dashed ellipse at the center of Figure 2. The photochromic cycle may be monitored through the absorbance at 611 nm, whose temporal evolution is reported in Figure 2c. During continuous UV irradiation, the absorbance ($A_{MC}$) increases exponentially until a photostationary state is reached. When irradiation is interrupted, the absorbance decreases because MC is a metastable species that thermally relaxes back to the original SpO form. As a consequence, the blue coloration gradually disappears. If the merocyanine population consists of multiple conformers characterized by different kinetic stabilities, the bleaching process can be represented as a superposition of exponential decays:

(7) $$A_{MC}(t)=\sum_{k=1}^{N_{C}}w_{k}^{\prime }e^{-t/\tau_{∆,k}}$$

In Equation (7), it is assumed that each conformer has its lifetime ($\tau_{∆,k}$ with $k=1,\ldots ,\;N_{C}$) and a corresponding weighting coefficient $w_{k}^{\prime }$. The determination of the optimal lifetime distribution can be achieved through the Maximum Entropy Method (MEM), which yields the least-biased distribution compatible with the experimental data [34,35,36,37,38,39]. The lifetime distributions obtained for MC dissolved in pure ethanol at different temperatures are shown in Figure 2d. Two clear trends emerge. First, increasing temperature shifts the distribution toward shorter lifetimes, indicating a faster thermal decay of the merocyanine population. Second, the distribution becomes broader, reflecting an increase in the diversity of kinetically distinguishable conformational states. The conformational distribution can also be modulated by chemical additives. As illustrated in Figure 2e, the addition of glycine produces both a narrowing of the lifetime distribution and an increase in the average lifetimes. This behavior is consistent with the formation of supramolecular interactions between glycine and the zwitterionic forms of the merocyanine, which selectively stabilize the conformational ensemble [40,41]. Because pure ethanol constitutes a chemically homogeneous solvent environment, the observed lifetime distributions can reasonably be attributed primarily to the conformational heterogeneity of the merocyanine itself rather than to environmental microheterogeneity. The results reported in Figure 2d,e therefore demonstrate that the conformational distribution of MC is highly sensitive to physicochemical variables such as temperature and molecular interactions. Most importantly, the distribution partitions the kinetic variable time into a set of weighted lifetimes, thereby generating a graded representation of molecular behavior. In the language of fuzzy-set theory, the lifetime distribution acts as a context-dependent granulation of the temporal domain, with the weighting coefficients playing a role analogous to degrees of membership. Consequently, the MC conformational ensemble exhibits the essential characteristics of a molecular fuzzy set.

Any molecular fuzzy set encodes an amount of Fuzzy Information that can be quantified through the following expression of Fuzzy Entropy [42]:

(8) $$H=-\frac{1}{log(N_{C})}\sum_{k=1}^{N_{C}}\mu_{k}log(\mu_{k})$$

wherein

(9) $$\mu_{k}=\frac{w_{k}^{\prime }}{\sum_{k=1}^{N_{C}}w_{k}^{\prime }}$$

Our rationale is that, for a molecule distributed among many conformers, the probability of finding the molecule in a given conformational state may be used as an operational measure of the degree to which that conformer participates in the overall molecular identity. In this restricted sense, the conformational populations provide a physically motivate realization of membership values. We therefore employ probabilities as quantitative surrogates for memberships, rather than asserting that memberships and probabilities are universally identical concepts.realization of membership values. We therefore employ probabilities as quantitative surrogates for memberships, rather than asserting that memberships and probabilities are universally identical concepts.

H can assume any real value included between 0 and 1, and its unit is called *fit*. The values of H for the conformational distributions shown in Figure 2d,e are listed in Table 1.

The data in Table 1 reveal that temperature increases the amount of fuzzy information encoded by the merocyanine conformational ensemble. In pure ethanol, H rises from 0.24 fits at 292 K to 0.74 fits at 308 K, indicating a progressive expansion of the accessible conformational space. Temperature therefore acts as a *fuzzy-information decoder*, increasing the diversity of populated conformational states. In contrast, glycine reduces the value of H to values below 0.27 fits over the entire temperature range investigated. The interaction with glycine thus acts as a *fuzzy-information encoder*, restricting the conformational freedom of the merocyanine and reducing the diversity of the populated states.

Since conformational ensembles can be described simultaneously as molecular fuzzy sets and thermalized quantum mixed states, their modulation through physicochemical parameters corresponds to the manipulation of an entire population of molecular states rather than of a single molecular configuration. From this perspective, variations in temperature, solvent composition, or supramolecular interactions alter the probability distribution of the conformational ensemble, thereby affecting multiple conformational states simultaneously. As a result, the experimentally observed molecular properties emerge from the collective contribution of a statistically populated ensemble of conformers rather than from any individual molecular structure [22]. 

### 3.2. Experimental Evidence Supporting the Fuzzy-Set Description of Biopolymers

Conformations play important functional roles in biopolymers, such as proteins, DNA, and RNA.

#### 3.2.1. Proteins

In 1894, Hermann Emil Fischer proposed the influential *lock-and-key* model to explain the remarkable selectivity of enzymes toward their substrates [44]. According to this model, efficient catalysis requires a precise geometric complementarity between the enzyme active site and the substrate, analogous to the fit between a lock and its key. This concept laid the foundation for the classical structure-function paradigm, claiming that biological activity arises from a unique three-dimensional organization of amino acid residues and cofactors. The paradigm was later reinforced by Anfinsen’s thermodynamic hypothesis, which established the sequence of amino acids as the primary determinant of a protein’s native structure and, consequently, its biological function [45,46]. In its simplest formulation, this view may be summarized as a “unique sequence-unique structure-unique function” relationship.

**Figure 3:**
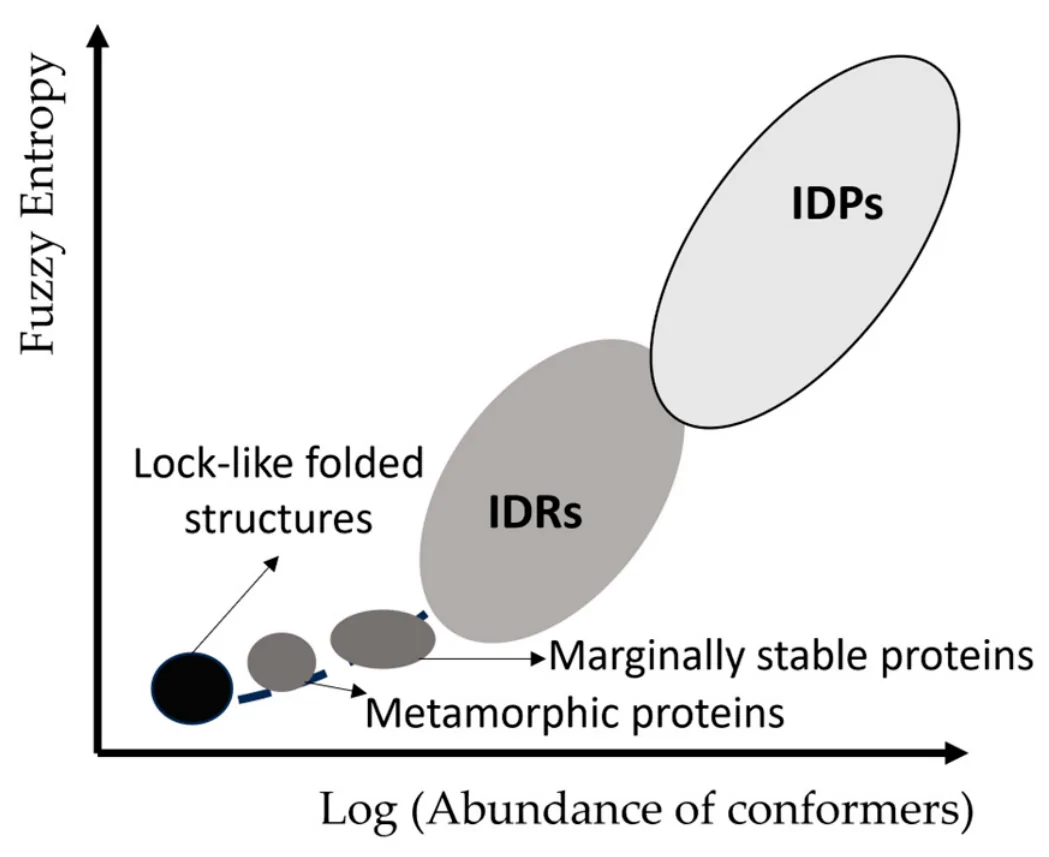
Trend of Fuzzy Entropy (Equation (8)) that depends on both the number of accessible conformers and the distribution of their populations. Maximum entropy is obtained when the conformers are populated as uniformly as possible, whereas entropy decreases when the population becomes concentrated in a limited number of states.

Over the last three decades, however, advances in nuclear magnetic resonance (NMR) spectroscopy, single-molecule techniques, time-resolved spectroscopies, cryo-electron microscopy, and computational modeling have revealed a much more dynamic picture of proteins in their native environments. Rather than behaving as rigid molecular objects, proteins populate ensembles of interconverting conformational states characterized by different free energies and populations [47]. Protein function is therefore increasingly understood as an emergent property of a conformational distribution rather than of a single static structure. This ensemble view gained further support from the discovery of intrinsically disordered proteins (IDPs) and intrinsically disordered regions (IDRs), which demonstrated that biological activity can be preserved, and often enhanced, in the absence of a stable tertiary structure [48,49,50,51]. Consequently, the traditional structure-function paradigm has evolved into a protein structure-function continuum [52,53,54], spanning a broad spectrum of conformational complexity (Figure 3). Within the framework proposed here, this continuum can be quantified through the concept of Fuzzy Entropy (Equation (7)), which increases with the abundance and diversity of accessible conformational states [18]. At one extreme of the continuum are highly structured proteins whose behavior approximates the classical lock-and-key model. These proteins occupy a relatively small conformational space and therefore exhibit low fuzzy entropy. Note that even the most stable ordered proteins are not completely crystal-like, and instead the bottoms of their folding funnels (i.e., the lowest parts of their potential energy landscapes) are not smoothed but rugged, reflecting significant conformational flexibility [55]. A modest increase in fuzzy entropy is observed for metamorphic proteins, which reversibly interconvert between two or more distinct native folds while retaining biological activity [56]. Higher values occur in marginally stable proteins, whose folded and unfolded states are separated by small free-energy differences, allowing environmental fluctuations to strongly influence the conformational population [57]. The conformational complexity increases further in proteins containing IDRs. These proteins are composed of structural modules with different degrees of order and disorder, including independently foldable domains (*foldons*), *inducible foldons* that become structured upon partner binding, *morphing inducible foldons* capable of adopting alternative conformations, *non-foldons* that remain disordered yet functional, *semi-foldons* that are only partially folded, and *unfoldons* that require local unfolding to become active [58,59,60]. Because these proteins continually explore multiple structural states, they possess substantially larger conformational repertoires and correspondingly higher fuzzy entropy values. At the opposite extreme of the continuum are intrinsically disordered proteins (IDPs), which represent the highest level of conformational fuzziness. These proteins lack a unique folded state under physiological conditions and are instead best described as highly heterogeneous ensembles of rapidly interconverting conformations [61,62,63,64,65]. Their existence challenges the strict interpretation of Anfinsen’s dogma and demonstrates that biological function can emerge directly from conformational diversity rather than from structural rigidity.

From the perspective developed in this work, the progression from rigid proteins to IDPs can be interpreted as a progressive increase in molecular fuzziness. As the number of accessible conformational states grows, proteins acquire enhanced capacities for adaptation, context sensitivity, and information processing. In this sense, Figure 3 can be viewed as a map of increasing fuzzy complexity within the protein world. This idea aligns with recent research suggesting that the classical “one sequence → one structure → one function” lock-and-key paradigm for proteins should be extended to a new conceptual model sequence → dynamics → conformational ensembles → context-dependent behavior [66], which implicitly accounts for molecular fuzziness.

Conformational heterogeneity is a major determinant of protein multifunctionality, promiscuity, and adaptability [67,68,69]. Because conformational distributions depend strongly on the physicochemical context, the same protein may exhibit distinct functional behaviors under different environmental conditions. This principle underlies the phenomenon of protein moonlighting, whereby a single polypeptide chain performs multiple biological functions through context-dependent shifts in its conformational ensemble. These shifts may involve subtle local rearrangements, large-scale domain motions, or major alterations in tertiary and quaternary structure [70,71].

The functional relevance of conformational fuzziness is particularly evident in IDPs and IDRs. Owing to their structural flexibility, these proteins participate in a wide range of biological processes, including gene regulation, signal transduction, translation, cell-cycle control, circadian regulation, and cellular differentiation [60]. Their ability to sample many conformational states allows them to interact with multiple partners, function as signaling hubs, and coordinate complex regulatory networks [68,72,73]. Protein conformational fuzziness is deeply interconnected with post-translational modifications (PTMs), and these two intertwined mechanisms are used by cells to achieve highly versatile, context-dependent biological signaling [74]. Notably, IDRs serve as primary targets for many cellular PTMs [75,76,77,78], and PTMs act as precise molecular toggles that reshape their dynamic conformational landscapes, acting through several distinct mechanisms, such as conditional folding, ensemble shifting, and regulation of interactability. Overall, fuzziness and PTMs represent a mighty duo. Cells must recognize thousands of specific targets using a limited genome. Fuzziness allows a protein to adapt to different partners, while rapid, enzymatically reversible PTMs ensure that this multi-target binding is strictly regulated by the cellular context. Traditional allostery requires structural shifts propagated through a rigid backbone. In fuzzy proteins, a PTM at a distant regulatory site can alter the overall entropy or flexibility of the main binding interface instead, effectively tuning affinity without needing a static shape change. PTMs frequently lock fuzzy domains into transient self-binding patterns. This creates an autoinhibitory shield that prevents improper downstream cascades until an explicit upstream signal removes or alters the modifications.

Furthermore, many IDPs drive the formation of biomolecular condensates through liquid–liquid phase separation (LLPS), thereby generating a multitude of dynamic and reversible intracellular compartments (biomolecular condensates (BCs) or membraneless organelles (MLOs)), which regulate biochemical reactions and cellular organization [73,79,80], and which represent an important illustrative example of large fuzzy ensembles ranging in size from 10 nm to over 1 µm. In fact, it was emphasized that BCs/MLOs, being an intriguing form of disorder-based protein assemblages typically formed without noticeable structural changes or ordering of their constituent IDPs/IDRs, are characterized by a highly dynamic nature defining their liquid-like appearance, indicating that supramolecular fuzziness is crucial for many aspects of BC/MLO biogenesis, stability, and functionality [81].

Conformational diversity also plays a fundamental role in immune recognition. Despite possessing a finite repertoire of receptors and antibodies, the immune system is capable of recognizing an almost unlimited diversity of antigens. This remarkable capability arises, in part, from the structural plasticity of immune proteins [82]. Different antigens may preferentially bind distinct members of a receptor conformational ensemble, while receptor flexibility promotes cross-reactivity and multispecificity, thereby increasing the adaptive potential of the immune response [83]. Consistent with this interpretation, intrinsic disorder is particularly prevalent among proteins involved in innate antiviral immunity, where structural flexibility provides an evolutionary advantage in recognizing rapidly evolving pathogens [84]. Likewise, substantial mobility and disorder within antigen-binding interfaces are now recognized as compatible with, and frequently beneficial for, efficient immunochemical recognition [85].

Taken together, these observations indicate that proteins are not adequately described as static molecular entities. Rather, they should be viewed as context-dependent molecular fuzzy sets, whose conformational distributions encode information, mediate environmental responsiveness, and enable adaptive biological functions. The protein world therefore provides compelling experimental support for the central thesis of this work: conformational complexity and molecular fuzziness are intrinsically linked manifestations of the same underlying physical reality.

#### 3.2.2. Nucleic Acids (NAs): Fuzzy Molecular View

For many, DNA and RNA (which are simple linear polymers consisting of only four major types of subunits) are associated with the iconic, textbook representations, where DNA is depicted as a double helix (“twisted ladder”) consisting of two intertwined, antiparallel, complementary strings of polynucleotide chain, and RNA, being associated with tRNA, is seen as a cloverleaf or an inverted L-shaped 3D structure. However, these “iconic” images are misleading. In fact, in addition to the canonical B-form (which, being the most common form in cells, represents a right-handed helix with a wide major groove and a narrow minor groove), DNA can exist in several structurally different forms, such as hairpin, cruciform, right-handed A-DNA, left-handed Z-DNA, triple-stranded H-DNA (or DNA triplex), and even four-stranded G-quadruplex and i-motif [86]. Furthermore, in its single-stranded form, DNA molecule (ssDNA) is not merely a linear sequence of nucleotides but is highly flexible and capable of folding into various stable secondary structure elements in a condition-dependent manner and can even be organized in a tertiary structure (see below). 

Similarly, the rigid L-shaped form of tRNA serves as a very poor structural representation of RNA in general, as RNA structure is incredibly diverse and dynamic, whereas tRNA is highly specialized and conserved. In fact, non-tRNA RNAs, such as messenger RNAs (mRNAs), and various non-coding RNAs (ncRNAs), such as ribosomal RNAs (rRNAs), small nuclear RNAs (snRNAs), small nucleolar RNAs (snoRNAs), and long non-coding RNAs (lncRNAs), adopt vastly different structural forms [87]. Finally, the genome of some viruses represents double-stranded RNAs (dsRNAs), i.e., RNAs with two complementary strands, and dsRNAs are generated in host cells during viral replication (including both RNA and DNA viruses) [88]. Furthermore, in various pathophysiological states, various dsRNAs can be produced from endogenous sources, such as retroelements and mitochondrial DNA [88].

Coming back to their iconic representations, the structural distinctions between DNA and tRNA directly determine their biological roles. The rigid, double-helical, “twisted ladder” configuration of DNA is optimized for preserving and protecting genetic information over the long term. Conversely, the intricate 3D structure enables the “adapter” function of tRNA, allowing it to precisely bridge mRNA codons with corresponding amino acids during protein synthesis. These structures are formed and stabilized by very different mechanisms. A double helix of DNA is stabilized by base pairing (formation of hydrogen bonds between complementary bases, Adenine-Thymine (AT) with two bonds and Guanine-Cytosine (GC) with three bonds) between the complementary strands and base stacking interactions, where the flat aromatic bases stack on top of each other, providing significant contributions to DNA stability through hydrophobic interactions, van der Waals forces, and pi-stacking [89]. On the other hand, a complex 3D structure of tRNA (as well as of any single-stranded RNA (ssRNA) and, as a matter of fact, of the single-stranded DNA (ssDNA) as well) is formed by the intramolecular folding of a single polynucleotide chain back on itself through internal base pairing [90]. 

Although the formation of all these structures is thermodynamically driven (i.e., at given conditions, a nucleic acid is organized into the most thermodynamically stable configuration), the mechanisms are different for single-stranded and double-stranded nucleic acids [91]. Double-stranded nucleic acid structures are formed through a two-step intermolecular annealing process known as hybridization or zippering. It begins with nucleation, where a small, transient, and unstable nucleus of complementary base pairs forms, followed by rapid zippering of the remaining pairs [92]. This process is driven by free-energy minimization, resulting in a highly stable, uniform helical structure that resists environmental degradation [93]. Importantly, dsRNAs were shown to possess mechanic properties similar to those of dsDNAs [94]. Although some secondary structure elements (e.g., hairpin, loops) can be formed within the DNA single strands, they are considered as unproductive traps that significantly reduce hybridization rates by masking complementary sequences and increasing the kinetic barrier for the interchain binding [95,96,97]. One should also keep in mind that various ssDNA structures formed via intrastrand pairing, being known as “folded DNA”, are generated in several cellular processes and involved in important biological functions and therefore can be beneficial as well [98]. 

Single-stranded nucleic acids (ssNAs) can form complex, functional 3D structures due to their ability to fold back on themselves via intramolecular base pairing, leading to the formation of diverse structural elements, such as hairpins, stems, loops, and bulges, which are primarily stabilized by hydrogen bonds between bases (A-U/T and G-C) [99]. Much like proteins, ssNAs fold through a complex, hierarchical process. Intramolecular Watson–Crick and wobble base pairing first form secondary structure elements, such as helices, hairpins, and loops. Next, distant secondary structure elements are “glued” together in 3D space by tertiary contacts, such as kissing loops and pseudoknots, thereby forming 3D tertiary structures. This folding is driven by base pairing, base stacking, ion interactions (particularly, cation-driven stabilization of the phosphate backbone, as nucleic acids are highly charged polyelectrolytes, whose negatively charged backbone causes intense internal repulsion), noncanonical interactions, and sometimes protein assistance, where proteins act as chaperones, guiding or stabilizing specific folds. The process often occurs co-transcriptionally, navigating intricate energy landscapes where the molecule may encounter kinetic traps before achieving its functional conformation [100].

Since the backbone of nucleic acids is highly anionic due to the negatively charged phosphates, ssDNA and ssRNA cannot spontaneously form stable tertiary structures in aqueous media without the presence of metal ions (counterions, especially Mg^2+^) to screen these charges, as the repulsive forces between them prevent the close packing of helices required for tertiary folding. Due to a strong electrostatic repulsion created by the high negative charge of the RNA phosphate backbone these molecules cannot collapse into a compact, functional 3D state. However, they can temporarily form basic secondary structures (such as simple hairpins or duplexes). As a result, in the absence of counterions, ssNAss exist as highly flexible, extended, disordered, random coil-like conformational ensembles [101,102,103,104,105,106]. Although some rare “ultra-stable” RNA structures (e.g., the UUCG stem-loop) that utilize a dynamic, “flexible” mechanism to rapidly select folding pathways and resist unfolding were recently identified, these structures still rely on environmental factors for stabilization [107].

Structures of nucleic acids, being inherently dynamic entities, are highly dependent on binding to specific interaction partners, most noticeably proteins [108]. A major role in molecular association of proteins with nucleic acids is played by electrostatic interactions between positively charged protein side chains and negatively charged phosphates [109]. These dynamic interactions are fundamental to biological regulation and genomic organization, playing a number of crucial roles in a wide set of biological processes, ranging from genome replication and protection to gene expression, and from transcription and splicing to protein translation and immune response [110,111]. For example, proteins can modify a helical configuration of dsDNA by either unwinding or overwinding it [112,113,114], with the corresponding bending, stretching, and twisting deformations of DNA induced by protein binding being crucial for numerous cellular processes [115]. RNAs are targeted by more than 2000 RNA-binding proteins (RBPs), which use different structures and mechanisms to bind to and regulate structurally very diverse RNAs [116]. Similar to protein-DNA interaction, binding of an RBP to RNA is typically associated with the reciprocal structural adjustments to achieve high specificity and stable complex form [109]. The fact that many NA-binding proteins are intrinsically disordered or contain long functional intrinsically disordered regions is well established [117,118,119,120,121,122,123,124,125,126,127,128,129,130,131,132]. In fact, functionally important disorder is found in a wide range of proteins, such as transcription factors [133,134,135,136,137,138,139,140], nucleosomal proteins (histones) [141], ribosomal proteins [142], spliceosomal proteins [143,144,145], and mitochondrial enzymatic machines [146]. Furthermore, intrinsic disorder in RNA- and DNA-binding proteins is considered an important prerequisite for LLPS, a crucial process linked recently to the biogenesis of various MLOs and BCs [147,148,149,150,151,152,153,154,155,156,157]. It was also indicated that an interplay between the disordered regions of RNAs and protein IDRs is important for efficient LLPS [158] and that LLPS can be further modulated by RNA modifications, such as N6-methyladenosine (m6A) modification of mRNA [159].

#### 3.2.3. Fuzziness of DNA Supramolecular Structure

Despite its extraordinary length, approximately two meters in a human cell, DNA is compacted within a nucleus only a few micrometers in diameter through a hierarchical and highly organized architecture. DNA wraps around histone proteins to form nucleosomes; nucleosomes assemble into chromatin fibers, which in turn fold into chromosomes [160]. This three-dimensional organization is far more than a packaging strategy: it is a fundamental determinant of gene activity. Processes such as gene transcription, replication, genome maintenance, and gene regulation depend critically on the spatial arrangement and dynamic remodeling of chromatin [161,162]. From the perspective developed in this work, DNA and chromatin provide another compelling example of how biological systems exploit molecular fuzziness. Genes should not always be regarded as discrete entities with rigid boundaries. Coding sequences may overlap, share genomic regions with adjacent genes, or even be completely embedded within other genes, giving rise to nested genetic architectures [163,164]. Such observations challenge purely binary views of genomic organization and suggest that genes can often be interpreted as fuzzy sets whose boundaries and functional relevance depend on biological context [165]. This interpretation is reinforced by the fact that gene activity is rarely an all-or-none phenomenon. Rather than existing simply in ON or OFF states, genes exhibit a continuum of expression levels that emerge from the interaction between chromatin structure, transcription factors, regulatory RNAs, and the transcriptional machinery [166,167]. Consequently, gene expression is more appropriately described as a graded variable than as a binary event. Within the conceptual framework proposed in this work, the regulatory network comprising chromatin organization, transcription factors, regulatory RNAs, and transcriptional machinery can be interpreted as exhibiting functional features reminiscent of a fuzzy inference system, whereby multiple contextual inputs contribute with different weights to the generation of graded transcriptional outputs. We present this as an explanatory analogy intended to illuminate the continuum-like nature of gene regulation rather than as an established mechanistic model of chromatin function. Euchromatic regions correspond to conditions that yield high firing strengths for transcriptional rules because they permit access to transcription factors and RNA polymerase. Conversely, heterochromatic regions correspond to conditions with firing strengths close to zero, as chromatin compaction severely limits access to the transcriptional machinery. The structural organization of chromatin is determined by the interplay between physicochemical conditions and epigenetic events [168,169]. Factors such as molecular crowding, ionic strength, protein-DNA interactions, DNA methylation, histone modifications, and chromatin-remodeling activities continuously alter chromatin accessibility. From a fuzzy-systems perspective, these variables can be interpreted as inputs to a chromatin-based inference engine. Rather than producing a binary outcome of “gene active” versus “gene inactive,” chromatin integrates multiple molecular signals and generates graded levels of transcriptional potential. Under this vision, DNA is not a passive repository of genetic information but a highly dynamic repository of potential propositions. Chromatin transforms these propositions into context-dependent fuzzy rules whose firing strengths are determined by epigenetic state, regulatory interactions, and environmental signals. Gene expression is therefore the defuzzified output of a distributed macromolecular inference engine, making the central dogma of biology less a rigid flow of information and more a process of continuous biological reasoning.

#### 3.2.4. RNA

RNA is another major carrier of chemically encoded intracellular information. Like intrinsically disordered proteins, RNA is an inherently flexible biopolymer capable of adopting multiple conformations in response to changes in its physicochemical environment. This remarkable structural plasticity underlies an extensive functional repertoire that extends far beyond its classical role as an intermediary between DNA and proteins. Indeed, RNA molecules can regulate gene expression, recognize molecular targets, organize supramolecular assemblies, and even catalyze chemical reactions in the form of ribozymes [170]. RNA structure is highly dynamic, and both secondary and tertiary conformations play essential roles in controlling cellular processes. Among these, RNA secondary structure is a major determinant of alternative pre-mRNA splicing [171], a mechanism through which multiple mature mRNA isoforms are generated from the same primary transcript. Alternative splicing contributes substantially to proteomic complexity and helps explain how a relatively limited number of genes can produce a much larger repertoire of proteins. Through selective intron removal and exon joining, a single transcriptional unit can give rise to multiple protein variants characterized by distinct structural and functional properties. From the perspective developed in this work, alternative splicing represents a clear manifestation of molecular fuzziness. Rather than encoding a unique and predetermined outcome, a gene can generate a spectrum of transcript variants whose relative abundances depend on cellular conditions. The primary transcript may therefore be regarded as a molecular system containing several potential expression pathways. The spliceosome, together with RNA-binding proteins and regulatory factors, integrates multiple contextual signals and selectively promotes particular splicing outcomes, working as a fuzzy inference process in which the pre-mRNA sequence is a collection of molecular fuzzy sets. Consequently, the resulting transcript population is not a single discrete product but a weighted ensemble of mRNA isoforms whose composition continuously varies with developmental stage, cell type, metabolic conditions, and environmental stimuli. 

This interpretation is reinforced by the observation that alternative splicing is strongly associated with protein intrinsic disorder [172]. Polypeptide segments affected by alternative splicing are most often intrinsically disordered. Because these regions do not contribute to rigid structural cores, they can be inserted, removed, or modified without severely compromising overall protein stability. As a result, alternative splicing can diversify regulatory and interaction capabilities while preserving protein viability. Intrinsic disorder therefore provides an evolutionary framework through which alternative splicing expands functional diversity, regulatory complexity, and phenotypic adaptability [172]. The interplay between RNA structural flexibility, alternative splicing, and protein intrinsic disorder creates a hierarchical cascade of molecular fuzziness. Conformational heterogeneity at the RNA level generates diverse transcript populations, which, in turn, produce proteins characterized by distinct conformational landscapes and functional repertoires. In this way, biological information is not transmitted through a rigid sequence of deterministic steps but through successive layers of context-dependent molecular diversification.

The biological significance of RNA conformational flexibility extends beyond contemporary cellular systems. The ability of RNA molecules to store information while simultaneously performing catalytic functions has placed RNA at the center of theories concerning the origin of life. According to the RNA world hypothesis, the earliest biological systems were based on self-replicating RNA molecules capable of acting both as genetic materials and as catalysts [173]. The exceptional conformational versatility of RNA would have allowed a relatively small number of molecular species to perform a broad range of functions, thereby increasing the adaptive potential of primitive biochemical systems.

In this context, RNA and intrinsically disordered polypeptides may have acted synergistically during the earliest stages of biological evolution [174]. Several authors have proposed that primordial proteins were unlikely to resemble modern highly specialized enzymes. Instead, they may have consisted of flexible and structurally heterogeneous polypeptides with broad molecular-recognition and RNA-chaperone activities [175,176,177]. Such conformationally diverse molecules would have facilitated molecular interactions, promoted functional promiscuity, and enhanced the robustness of primitive biochemical networks.

From the perspective of molecular fuzziness, both RNA and intrinsically disordered proteins derive their extraordinary functional potential from their ability to explore large ensembles of conformational states. Their adaptive value stems not from structural rigidity but from the capacity to respond flexibly to changing physicochemical conditions. Consequently, the emergence and persistence of life may have depended, at least in part, on this conformational fuzziness, which provides a powerful mechanism for generating molecular versatility, functional innovation, and evolutionary adaptability. RNA therefore represents a particularly compelling example of how conformational complexity and molecular fuzziness are intimately connected throughout biological organization, from the earliest stages of life’s evolution to the sophisticated regulatory networks observed in modern cells.

## 4. Implications for Molecular Information Processing and Chemical AI

At the macroscopic and organismal levels, fuzzy logic has long been proposed as a useful framework for describing the human ability to process information expressed in natural language, owing to the conceptual similarities between fuzzy logic systems and certain structural and functional properties of the nervous system [28]. Studies in neuroscience have further suggested that biological structures operating at different hierarchical levels, including sensory receptor cells, afferent neurons, and even neocortical columns, may be interpreted in terms of fuzzy information processing [27,29]. The perspective developed in the present work extends this conceptual framework to the molecular scale by proposing that conformational distributions of molecules and biopolymers constitute physically instantiated molecular fuzzy sets. Within this framework, molecular fuzzy sets may be viewed as the elementary informational units of a physicochemical language whose syntax is defined by chemical reactions and whose semantics emerges from context-dependent molecular interactions. These considerations are consistent with Darwin’s principle that biological functions differ substantially “in form but not in kind” in the kingdoms of life [178]. Moreover, because molecular fuzzy sets can also be described as quantum mixed states, such information processing may be regarded as a form of quantum parallelism [22]. The fuzzy nature of biomolecules expands the conceptual boundaries of quantum biology. Biological systems may enhance their functional capabilities not only through quantum-coherent phenomena such as superposition, interference, tunneling, and entanglement [179], but also through the computational parallelism associated with quantum mixed states that implement fuzzy sets [22]. This property of living matter provides inspiration for the emerging field of Chemical AI [180,181,182], which seeks to emulate aspects of biological intelligence using molecules and macromolecules as computational hardware and physicochemical processes occurring in the liquid phase, including reactions, waves, diffusion, migration, convection, and advection, as software. In other words, the hardware and software of conventional AI are merged in the wetware of reactive chemical solutions maintained under thermodynamically nonequilibrium conditions, which is the characteristic physicochemical context of life. 

The molecular fuzziness described in this article provides a possible theoretical foundation for such systems. Because conformational ensembles inherently encode graded and context-dependent information, they may offer a natural route toward implementing forms of approximate reasoning directly in chemical media. Rather than representing information through binary states, molecular fuzzy sets operate through continuously varying populations of conformers that adapt to changes in their physicochemical context. Such features resemble some of the properties associated with adaptive, fault-tolerant, and context-aware forms of computation.

From a broader perspective, these ideas may have implications for research on Artificial General Intelligence (AGI), which is commonly defined as the capability of an artificial system to perform and transfer knowledge across a wide range of cognitive domains without task-specific reprogramming [183]. One of the major challenges in AGI research concerns the grounding of semantic information, namely the problem of connecting symbols and representations to meaningful interactions with the surrounding world. Living systems provide a remarkable example of such grounding because biological information is embedded in and constrained by molecular structure, physicochemical interactions, and environmental context.

The framework proposed here suggests that conformational ensembles may represent a molecular embodiment of context-dependent semantic information. In living cells, the biological significance of a molecule does not depend solely on its chemical composition but also on its conformational distribution and its interaction with the surrounding physicochemical environment. Consequently, meaning emerges from dynamic relationships among molecular states rather than from static symbolic representations. Whether analogous principles could be exploited in future artificial systems remains speculative; nevertheless, the possibility that semantic information may be implemented through adaptive molecular architectures represents an intriguing direction for Chemical AI and unconventional computing.

The concept of molecular fuzzy sets may provide a useful framework for interpreting molecular recognition processes. In many protein-ligand systems, binding does not arise from a unique molecular structure but from an ensemble of conformations whose populations are modulated by the interaction partner. In this context, molecular recognition can be interpreted as a shift in the membership distribution associated with the molecular fuzzy set [184,185,186,187,188].

Allosteric regulation provides another possible application of molecular fuzzy sets. Binding at one site modifies the population distribution of conformational states throughout the macromolecule, thereby altering the functional properties of distant sites. Such behavior can be naturally represented as changes in the membership values associated with the conformational ensemble [189,190,191].

At the systems level, molecular fuzzy sets may help describe cellular regulation, where biological functions often emerge from networks of interacting molecules characterized by overlapping states, graded responses, and strong context dependence. Because systems-level behavior arises from the collective organization of heterogeneous molecular states, the molecular fuzzy-set framework may provide a conceptual bridge between molecular complexity and emergent cellular functions [187,192,193,194].

Because many therapeutic targets populate multiple conformational states, molecular fuzzy sets may provide a useful language for structure-based drug design. Instead of targeting a single static structure, ligands may be designed to modulate distributions of conformers and thereby reshape functional behavior [184,187,195].

Conformational ensembles may also inspire adaptive molecular devices and responsive materials whose behavior depends continuously on environmental variables such as temperature, pH, ionic strength, or molecular composition. Such systems could exploit molecular fuzziness to generate graded responses to external stimuli [196,197,198,199].

In summary, the interpretation of conformational ensembles as molecular fuzzy sets extends the concept of fuzzy information processing from the cellular and organismal levels down to the molecular scale. Beyond their possible relevance to Chemical AI, molecular fuzzy sets may have applications in several areas of chemistry and biology, including molecular recognition, allosteric regulation, systems biology, drug discovery, synthetic biology, and adaptive materials. In all these cases, functional behavior emerges from distributions of conformational states rather than from unique molecular structures. The fuzzy-set framework provides a unified conceptual language for describing such phenomena in terms of graded participation and context-dependent molecular identity.

## 5. Conclusions

The evidence reviewed in this article indicates that molecular conformations are not merely structural details of biomolecules but fundamental determinants of biological function. Proteins, DNA, RNA, and many smaller molecular systems exist as dynamic ensembles of interconverting conformations whose populations depend on the surrounding physicochemical context. This conformational variability provides living systems with adaptability, multifunctionality, responsiveness, and robustness at the molecular, intracellular and cellular levels.

The central thesis of this work is that conformational ensembles are chemical instantiations of fuzzy sets. Just as fuzzy sets represent concepts through graded memberships rather than rigid boundaries, conformational distributions describe molecular states through weighted populations rather than unique structures. Within this framework, the physicochemical context determines the relative populations of conformers, thereby modulating the functional properties of the molecular system. Molecular identity therefore emerges not exclusively from chemical composition and connectivity but also from the distribution of accessible conformational states and their context-dependent dynamics.

Several examples support this interpretation. The conformational distributions of photochromic merocyanines demonstrate that molecular ensembles can granulate physicochemical variables and encode measurable amounts of fuzzy information. Likewise, modern molecular biology increasingly recognizes that proteins, DNA, chromatin, and RNA operate through ensembles of structures rather than through single static conformations. Intrinsically disordered proteins, alternative DNA and RNA conformations, chromatin remodeling, and context-dependent gene regulation all illustrate how biological functions emerge from conformational heterogeneity and dynamic structural plasticity.

Viewing conformational distributions as molecular fuzzy sets provides a unified conceptual framework linking conformational chemistry, molecular biophysics, information theory, and fuzzy logic. In this perspective, chemical reactions are not only transformations of matter and energy but also processes that modify, transmit, and process fuzzy information encoded in molecular ensembles. Conformational complexity and molecular fuzziness thus appear as complementary manifestations of the same underlying physicochemical phenomenon.

The practical relevance of fuzzy information processing is supported by the long-standing success of fuzzy logic in engineering applications that require adaptive behavior under conditions of uncertainty and nonlinearity. Fuzzy controllers have been successfully applied to problems ranging from the stabilization of inverted pendulums to autonomous guidance systems, pattern recognition, and the control of complex dynamical processes. Such achievements demonstrate the effectiveness of fuzzy reasoning in environments characterized by incomplete information, context dependence, and continuously changing conditions, features that are equally characteristic of biological systems.

The framework proposed here also suggests new directions for chemistry-inspired computing. Because conformational ensembles may be described simultaneously as molecular fuzzy sets and thermalized quantum mixed states, they provide a natural substrate for implementing parallel and context-dependent forms of information processing in molecular systems. This observation may contribute to the development of Chemical Artificial Intelligence (CAI), an emerging research field that seeks to exploit molecules, supramolecular systems, and reactive chemical media as computational platforms. In such systems, information is embodied in molecular structure and dynamics, while computation emerges from physicochemical processes occurring under nonequilibrium conditions.

More broadly, the concept of molecular fuzziness raises fundamental questions regarding the physical basis of meaning and information in living systems. Biological molecules do not simply store information; through their conformational ensembles they interpret, modulate, and respond to environmental conditions in a context-sensitive manner. Understanding this relationship between conformational complexity, fuzziness, and biological functionality may therefore provide new insights into the mechanisms of cellular organization, adaptation, cognition, and evolution.

Like any emerging conceptual framework, the molecular fuzzy-set approach possesses both strengths and limitations that should be considered when evaluating its scientific scope and applicability.

First, the molecular fuzzy-set framework should be regarded as a complementary conceptual layer built upon established theories of quantum mechanics, statistical thermodynamics, molecular dynamics, and systems biology rather than as a replacement for them. Its principal strength lies in its ability to describe molecular identity and function as distributed, graded, and context-dependent properties, particularly in systems characterized by conformational heterogeneity, structural plasticity, and multifunctionality. In such cases, the framework offers a language for representing gradual transitions between molecular states and for connecting molecular structure with ensemble-level functionality.

Nevertheless, some limitations remain. The assignment of membership values to molecular conformers remains partly model-dependent, and the relationship between statistical populations and fuzzy memberships requires further theoretical refinement. Moreover, the framework is currently interpretative rather than predictive, and direct experimental quantification of molecular fuzziness remains challenging. Future research should therefore aim to establish more rigorous links between fuzzy-set descriptors and statistical-mechanical quantities such as conformational probabilities, free-energy landscapes, entropy, and quantum density matrices. Equally important will be the identification of experimentally accessible observables capable of characterizing molecular fuzziness and assessing its functional significance.

In conclusion, conformational chemistry appears to offer a natural physical realization of fuzzy-set theory at the molecular scale. Whether regarded as a descriptive framework, a source of novel theoretical insights, or a basis for future applications in molecular information processing, the fuzzy-set perspective highlights the fundamentally distributed and context-dependent nature of biomolecular organization. The recognition that biomolecules function through context-dependent conformational ensembles not only deepens our understanding of living matter but also opens new opportunities for the development of unconventional computational paradigms inspired by the information-processing strategies evolved by life itself.

## Figures and Tables

**Table 1:** Values of Fuzzy Information H for MC determined from the conformational distributions plotted in Figure 2d,e, which refer to solutions in pure ethanol and in the presence of glycine, at different temperatures ^1^.

Temperature (K)	*H* in Pure Ethanol	*H* with Glycine
308	0.74	0.19
304	0.52	0.27
300	0.34	0.18
296	0.26	0.18
292	0.24	0.06

^1^ The original data have been published in [43].

## Data Availability

No new data were created or analyzed in this study. Data sharing is not applicable to this article.

## Abbreviations

The following abbreviations are used in this manuscript:

AGI	Artificial General Intelligence
AI	Artificial Intelligence
BC	Biomolecular Condensate
CAI	Chemical Artificial Intelligence
FLS	Fuzzy Logic System
IDP	Intrinsically Disordered Protein
IDR	Intrinsically Disordered Region
LLPS	Liquid–liquid Phase Separation
MC	Merocyanine
MEM	Maximum Entropy Method
MLO	Membrane-Less Organelle
NA	Nucleic Acid
PTM	Posttranslational Modification
RBP	RNA-Binding Protein
SpO	Spiro-oxazine
ssDNA	Single-stranded DNA
ssRNA	Single-stranded RNA
